# The Human Gut Phageome: Origins and Roles in the Human Gut Microbiome

**DOI:** 10.3389/fcimb.2021.643214

**Published:** 2021-06-04

**Authors:** Eleanor M. Townsend, Lucy Kelly, George Muscatt, Joshua D. Box, Nicole Hargraves, Daniel Lilley, Eleanor Jameson

**Affiliations:** ^1^ School of Life Sciences, The University of Warwick, Coventry, United Kingdom; ^2^ Warwick Medical School, The University of Warwick, Coventry, United Kingdom

**Keywords:** gut microbiome, bacteriophages, phage, metagenomics, isolation, biofilm, disease, diet

## Abstract

The investigation of the microbial populations of the human body, known as the microbiome, has led to a revolutionary field of science, and understanding of its impacts on human development and health. The majority of microbiome research to date has focussed on bacteria and other kingdoms of life, such as fungi. Trailing behind these is the interrogation of the gut viruses, specifically the phageome. Bacteriophages, viruses that infect bacterial hosts, are known to dictate the dynamics and diversity of bacterial populations in a number of ecosystems. However, the phageome of the human gut, while of apparent importance, remains an area of many unknowns. In this paper we discuss the role of bacteriophages within the human gut microbiome. We examine the methods used to study bacteriophage populations, how this evolved over time and what we now understand about the phageome. We review the phageome development in infancy, and factors that may influence phage populations in adult life. The role and action of the phageome is then discussed at both a biological-level, and in the broader context of human health and disease.

## Introduction

In the past decade, the importance of the human gut microbiome in both health and disease has come to light. It plays a role in the acquisition of nutrients, defence, and in a range of diseases, from gastrointestinal to neurological. Most of these microbiome studies focus solely on bacterial populations, with fungal, archaeal and viral communities often being neglected. However, it is of vital importance that the viruses of microbes, particularly bacteriophage populations, are not overlooked. These bacteriophages (phages), viruses that solely infect bacteria, are the most numerous viral component within the human body (Reyes et al., 2010; Minot et al., 2011), and are predicted to play a significant role in the maintenance of human health, through processes such as horizontal gene transfer (Camarillo-Guerrero et al., 2021).

Phages were first discovered in the early 1900’s simultaneously by d’Hérelle and Twort. At the time they were recognised as an alternative to antibiotic therapy (d’Herelle, 1918; d’Herelle, 1919; Twort, 1961), but faded into obscurity in Western medicine, before returning as a tool to improve human health in the face of the current antibiotic resistance crisis (Kortright et al., 2019).

While the majority of research has focussed on utilizing phages for therapeutic and biotechnology purposes, the field of analysing natural phage communities in the gut is relatively young and information on phages is often disregarded within microbial metagenome and metatranscriptome libraries. In order to effectively and safely administer phage therapy it is vital to understand natural phage interactions within the human microbiome. Typically, the investigation of the roles phages play in ecosystems has largely focused on marine and terrestrial environments, while the human body remains underexplored. In part, this may be due to issues arising from ethics surrounding samples of human origin and the difficulty of sampling the gut.

Bacteria are the most abundant cellular organisms in the gut. The gut is a nutritionally rich environment which allows bacterial concentrations of close to the theoretical maximum in the order of 10^7^ to 10^14^ cells, depending on the exact location (Sender et al., 2016). Therefore, there are an abundance of hosts available for phages to infect, which is of importance given the vast majority of viruses within the gut are phages. Together, the total community of phage populations is known as the phageome.

In this review we will discuss what is known about: how phages enter the body, how we study the phageome, its main components, and the important roles and functions it performs in both human health and disease.

## Methods to Identify and Characterize Phages in the Gut

**Figure 1:**
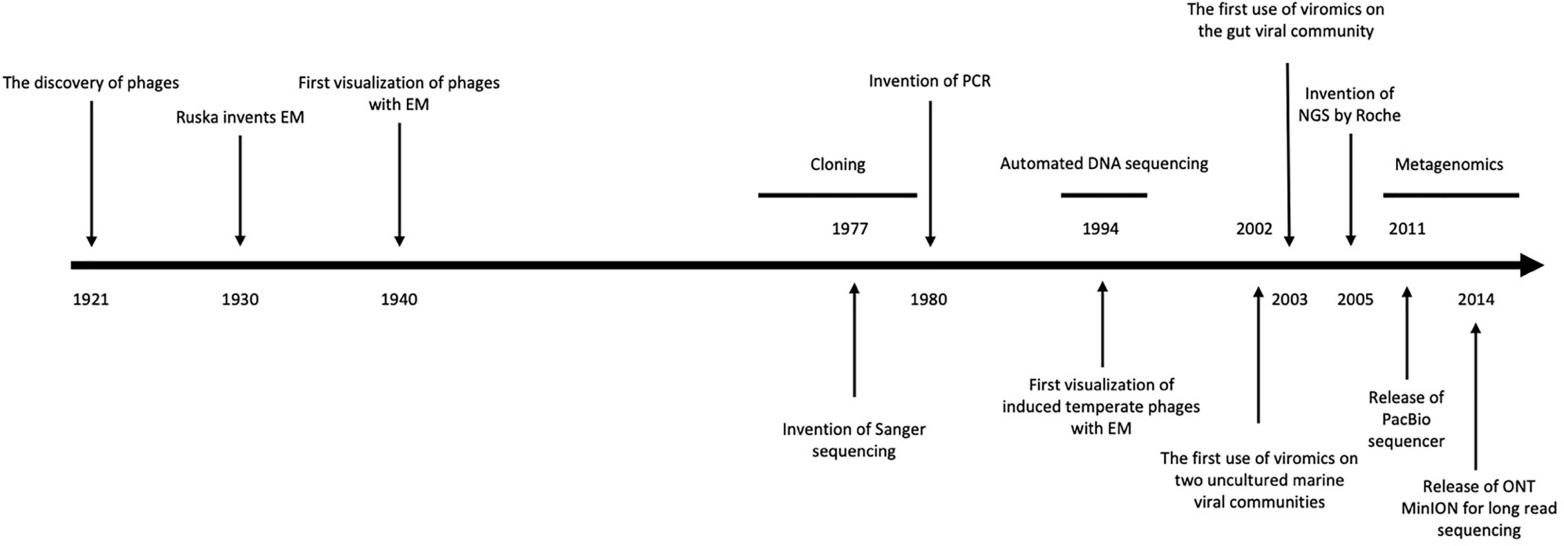
Timeline of methodological discoveries. A summarised timeline including the invention of key tools for the study of phages, from their discovery in 1921 to recent modern metagenomic sequencing technologies. Adapted from Mokili et al. (2012).

Since the early work of d’Hérelle (1921), many vital questions within phage biology, and specifically the role in ecosystems such as the human gut, remain unanswered. Techniques used for identifying and characterizing phages in the human gut can be understood from three perspectives: culture-*dependent* methods, culture-*independent* methods, and sequencing-based methods. Before the rise of next-generation sequencing in the last 15 years, culture-based methods were the primary approach for researching phage ecology, these rely on isolation techniques to extract virions for investigation (**Figure 1**) (Mokili et al., 2012).

### Culture-Dependent Methods

**Figure 2:**
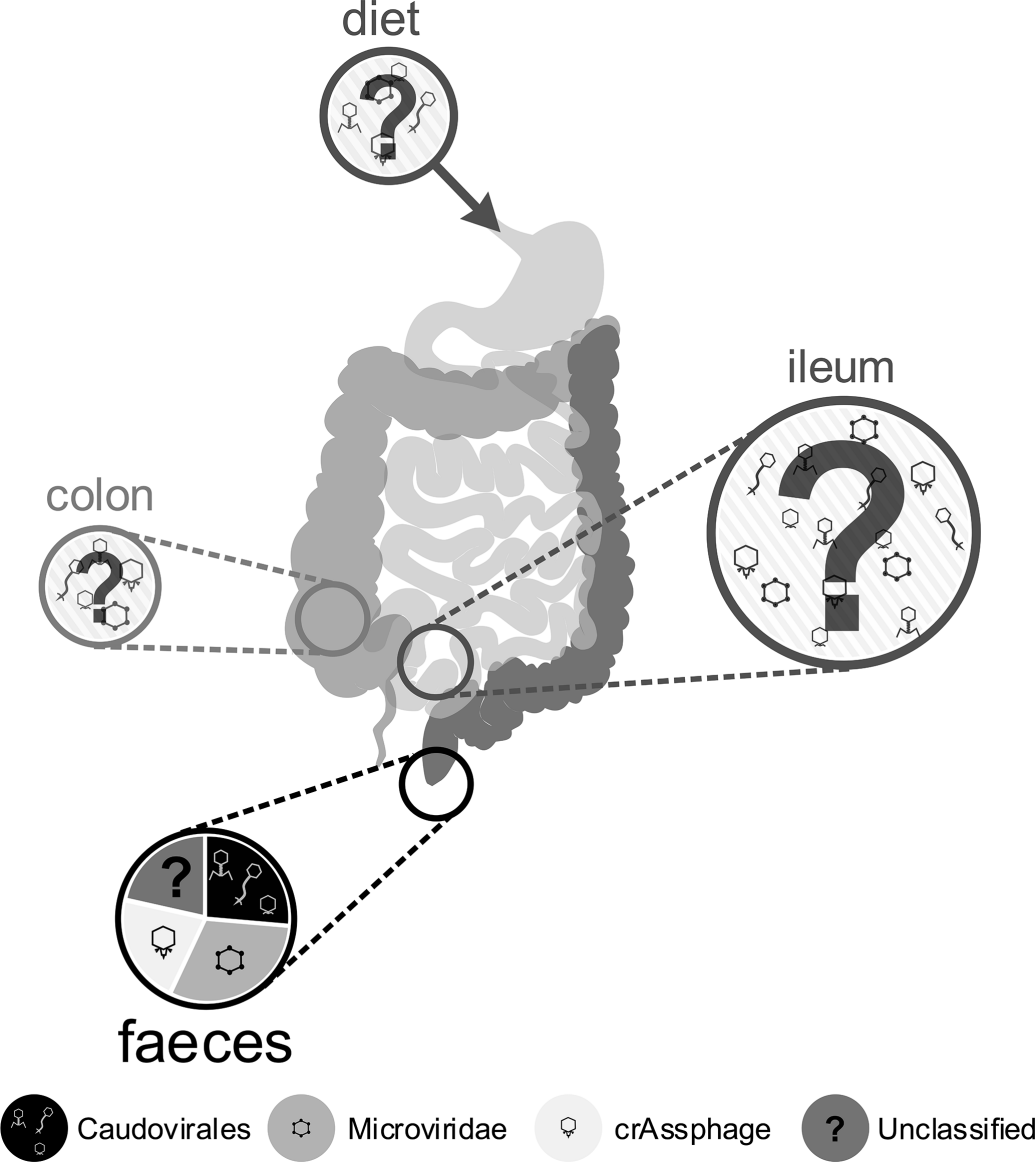
Schematic of the human gut, indicating unknown inputs to the gut phageome from diet and the environment, highlighting the largely unknown phage composition of the ileum and colon and indicating the relative proportions of phages in the best understood samples: faeces. Circle size provides a rough guide to the relative abundance of phage particles observed in different gut compartments (Zoetendal et al., 2012; Hoyles et al., 2014; Shkoporov and Hill, 2019), but current understanding is still lacking. Most research activity has focused on faecal samples, represented in the lowest pie chart, depicting the relative proportions of different phage groups identified through metagenome analysis, based on Shkoporov and Hill (2019).

For culture-based methods it is necessary to isolate virus-like particles (VLPs) from their complex *in situ* environment. This is often performed on faecal samples due to the ease and minimal cost in obtaining them. However, the *bacterial* community of the mucosa-associated microbiome is dominated by different phyla to those found in faeces (Lepage et al., 2005), so much so that it is compositionally distinct (Gillevet et al., 2010). Thus, sampling of faeces may select a combination of shed mucosal bacteria and nonadherent luminal populations (Eckburg et al., 2005). This sampling effect is likely to also affect the phage communities recovered. The relative understanding of phageome composition from faecal sampling is visualised in **Figure 2**, which highlights gaps in our knowledge from the acquisition of phages through diet, to the various positions of the digestive system. We suggest that future research efforts work towards a more holistic view of the human gut phageome, which undoubtedly relies on colonoscopies for the targeted sampling of the ileum and colon. This will facilitate the study of the phage populations in active luminal and mucosal-associated communities, in order to describe compositional differences in the respective phageomes, and to understand their possible *in situ* influence on the overall gut microbiome and the human host health.

Three approaches are taken to isolate phages from heterogeneous samples: extraction and purification of virus-like particles (VLPs), enrichment of phages in bacterial host cultures, and induction of temperate phages from lysogenised hosts. Extraction and purification of VLPs is the most common approach, which we describe below.

Following the suspension of faecal matter, intact VLPs can be separated from other microorganisms and free DNA in the faecal suspension by multiple methods, often used in combination (Thurber et al., 2009; Castro-Mejia et al., 2015; Kleiner et al., 2015). Conventional size-fractionation filtration to separate out phages introduces bias against larger VLPs, highlighting the need for novel methods (Serwer et al., 2009; Conceicao-Neto et al., 2015). The efficacy of VLP enrichment can be validated with staining and epifluorescence microscopy (see section *Culture-Independent Methods*). Gut-dwelling phages can subsequently be isolated using gut-specific bacterial strains to screen for infecting phages within the VLP fraction.

Isolating phages that infect bacteria cultured from the same environment has been readily applied to model hosts including *Escherichia coli* and *Bacteroides fragilis* (Gantzer et al., 2002). These can then be used to characterize aspects of phage-host biology, such as phage virulence and persistence. Further, isolation and cultivation is essential to characterize phage replication strategies and understand phage gene function, particularly given that we are limited in assigning functions to ~30% of phage genes (Silveira et al., 2020). While culture-based methods prove useful in isolating candidates for therapeutic and industrial applications (Bonilla et al., 2010; Mattila et al., 2015), the isolation of gut phages for ecological purposes is limited by host selection bias. In fact, it is challenging to culture gut bacterial strains or obtain those already cultured, with many sampling efforts focused on aerobic bacteria associated with disease from a small percentage of the human population.

To overcome this issue, recent progression in the field of culturomics (Lagier et al., 2012), which combines diversified culturing conditions (Lagier et al., 2015) with mass spectrometry (MALDI-TOF) and 16S rRNA sequencing, has helped to identify much of this bacterial dark matter of the gut, once thought to be uncultivable (Lagier et al., 2015). This technique has been used to add many gut bacterial isolates to the repertoire for isolating gut phages (Lagier et al., 2016). For example, the combination of culturing, enrichment and sequencing techniques has led to the discovery and isolation of the widespread human *CrAssphage* (see section *Identification of Novel Human Phages*) (Dutilh et al., 2014; Shkoporov et al., 2018a).

Temperate phages, integrated within their bacterial host genomes as prophages, also pose a challenge for isolation, but can be induced from lysogenised isolates using UV light (Furuse et al., 1983), mitomycin C, and heat treatment (Pelzek et al., 2013). Induced prophages can then be separated from their hosts using the same methods as for virulent phages. Non-lysogenised reporter host strains can then be used to verify temperate phage infectivity. There is debate over the relative importance of virulent and temperate phages in the gut (see section *Phage-Host Population Dynamics*), therefore the isolation of a range of phages with different replication strategies is key to testing these hypotheses.

In addition to characterizing aspects of phage-host interactions, concentrated phage populations can be further interrogated with flow cytometry (Brown et al., 2015), microscopy and molecular biology techniques (Castro-Mejia et al., 2015) (see section *Culture-Independent Methods*), or sequenced for further *in silico* analyses (see section *Metagenomics*). Culture-dependent methods of phage isolation and characterisation focus on individual phage functions and replication strategies. Despite this being vital for the investigation of phage populations and phage-host dynamics, culture-independent methods have facilitated the study of phageome diversity and function.

### Culture-Independent Methods

While the recent rise in culture-independent methods since the 1980s was due to the advent of Sanger sequencing, these methods arose decades earlier thanks to electron microscopy (EM) (**Figure 1**). Phages were first visualised with EM in the 1930s (Peankuch and Kausche, 1940; Ruska, 1940), which circumvented the magnification limitations of light microscopes, allowing for the visualisation of phages. Transmission EM (TEM) was used to observe that tailed phages were the most abundant VLPs in human faeces (Flewett et al., 1974), with recent microscopy indicating that the gut virome is almost exclusively represented by the order *Caudovirales* (Hoyles et al., 2014; Guerin et al., 2018). A large diversity of viral morphotypes have been visualised within individuals (Hoyles et al., 2014), with most VLPs representing phages. Microscopic techniques have also been used to enumerate gut phages (Lepage et al., 2008; Hoyles et al., 2014), however it has been suggested that TEM and staining could overestimate the true active phageome by up to 8 times (Kleiner et al., 2015). An evaluation of the staining and visualisation techniques that have led to a number of important discoveries is given in **Table 1**. The revelation of the high abundance of viruses, especially phages, in the human gut as observed by microscopy has paved the way for modern phage research.

**Table 1:** Microscopy techniques used to study the gut phageome, including the major advantages and limitations of each technique.

Technique	Example gut discoveries	Advantages	Limitations
TEM	Tailed phages were the most abundant VLPs in human faeces (Flewett et al., 1974).	Visualisation of phage morphology.	Biased towards identifying tailed phages due to potential loss of tail structures in sample preparation (Williamson et al., 2008).
	Faecal samples from patients were found to share no VLPs (Hoyles et al., 2014).		Limited to observations of morphologies.
			Time-consuming.
EFM	Up to 5.58 x 10^9^ VLPs were observed per gram of faeces (Hoyles et al., 2014).	Enumeration of VLPs in samples.	VLP counts are conservative estimates of true viral abundances, given the imprecision of visualising single fluorescent dots.
		Can validate viral purification procedures.	Loss of VLPs during preparation and filtration of samples, e.g. large VLPs of the order *Megavirales* (Colson et al., 2010).
		Greater accuracy and speed compared to TEM.	Viability of the VLP to infect and lyse bacterial cells is unknown.
			VLPs may be membrane vesicles, gene transfer agents or cell debris containing nucleic acids (Forterre et al., 2013).
phageFISH	The viability of VLPs can be determined through single cell dynamic measurements, as shown with marine phages (Allers et al., 2013).	PhageFISH is the only non-genetic method to implicate lytic, lysogenic, and chronic phage infection modes (Dang and Sullivan, 2014).	
CLSM	The complex microenvironment and spatiotemporal succession can be studied in multispecies biofilms, as shown with non-phage viruses (Røder et al., 2016).	Non-destructive sampling. Can be used to visualise the biofilm infection over time.	Limited to biofilms of bacterial species which can be fluorescently labelled.
CryoEM	Phage capsids of *Bortadella* phages were visualised at angstrom resolution, discovering unique protein folds, as shown with non-gut phages (Zhang et al., 2013).	Very high resolution.	Destructive sampling.

Modern molecular biology techniques reflect the biochemical, technological and high throughput advancements made over the past few decades. The use of which have resulted in breakthroughs such as the identification of toxin-antitoxin systems in *Lactobacillus johnsonii* prophages using recombinant protein gene expression (Denou et al., 2008). The use of molecular biology for gut phage research is evaluated in **Table 2**.

**Table 2:** Summary of molecular biology techniques used to study the gut phageome, including the major advantages and limitations of each technique.

Technique	Example gut discoveries	Advantages	Limitations
Electrophoresis	SDS-polyacrylamide gel electrophoresis on a CsCl fraction of human faeces isolated CrAss-like phages for subsequent mass spectrometry (Guerin et al., 2018).	Multiple viral populations can be separated from multispecies samples due to their differing capsid sizes.	No taxonomic information can be determined by electrophoresis alone.
Recombinant protein gene expression	Toxin-antitoxin systems were identified within prophages of *Lactobacillus johnsonii* (Denou et al., 2008).	Proteins can be overexpressed to obtain high titres for subsequent analysis.	Cloning of viral genes into heterologous host systems can be difficult given that viral genomes often encode modified nucleotides (Warren, 1980) and lethal genes (Wang et al., 2010).
			Expressed proteins may not be functional due to misfolding and incorrect modifications in expression host.
Microarrays	The new-born infant gut viral community was found to be dynamic (Breitbart et al., 2008).	High-throughput.	Incompatible with novel viral genomes as *a priori* sequence information is required to design probes.
	50% of the strain-specific DNA in *Lactobacillus johnsonii* was found to derive from prophages (Ventura et al., 2003).		Viral DNA amplification steps prior to microarray analysis can introduce bias, making relative abundances no longer reflect that of the sample studied.
Single-cell DNA sequencing	Sequencing of commensal gut bacteria can facilitate the identification of integrated prophages (Ventura et al., 2003; Denou et al., 2008).	Facilitates taxonomic investigation.	Requires isolation and cultivation of lytic phages and hosts of temperate phages.
		Can assemble viral genomes for viruses excluded from metagenomic approaches (Martinez-Hernandez et al., 2017).	No community-wide view.
qPCR	77% of faecal samples contained phages carrying at least one antibiotic resistance gene (Quiros et al., 2014).	Detection and quantification of specific genes in real time.	Sequence of target gene is required *a priori*.
	Longitudinal tracking of phage and bacterial hosts in the faeces of a mouse model system facilitated the study of predator-prey dynamics (Hsu et al., 2019).		Nonspecific binding of template can lead to amplification of off-target genes.
Viral tagging & flow cytometry	363 unique phage-host pairings were predicted, including many uncharacterised phages (Džunková et al., 2019).	Infer phage-host relationships.	Direct evidence of successful phage infection is not provided by attachment of phage and host cell.
		Culture-independent.	Different assay conditions can bias the phage-host pairings observed from the community.
		Flow cytometry has the facility to sort individual phage-host pairs for downstream sequencing etc.	

It is finally worth noting the use of whole animal models for investigating the factors that drive microbiome structure and function, and disease-state modelling of the human gut, e.g. inflammatory bowel diseases (see section *Role of Bacteriophages in Human Disease*). Animal models enable the experimental investigation of phage-host interactions, to develop hypotheses that connect alterations in the gut microbiome to disease. An extensive review of simple animal models of the gut microbiome was written by Douglas (2019). Murine models have been applied to a wide range of phage studies, including phage-host population dynamics over time (Hsu et al., 2019), changes in phage populations after antibiotic exposure (Modi et al., 2013), investigating the role and function of phage proteins (Miernikiewicz et al., 2016), and the abundance and activity of prophages in the gut (Kim and Bae, 2018). However, there has been much critique of the use of mice models to represent the human gut, discussed in Nguyen et al. (2015). Animal models have enabled hypothesis generation and testing, but due to differences in their physiology and natural microbiota, they should be used as a stepping-stone rather than as final evidence to inform human microbiome related medicine.

### Metagenomics

Metagenomics is the most recent development on the gut phageome methods timeline, facilitated by the advent of high-throughput sequencing (**Figure 1**). Metagenomics provides a snapshot of the DNA phage community: the populations that can be recovered, and the genes they encode. There are two favoured approaches to performing metagenomics: sequencing of the entire nucleic acid complement through *shotgun metagenomics*, and amplification of marker genes such as the 16S ribosomal RNA gene in *amplicon sequencing* (Jovel et al., 2016; Hayes et al., 2017). Given the absence of a universal viral marker gene, amplicon approaches are unviable for studying the phageome, although some group-specific markers do exist for estimations of phage diversity (Adriaenssens and Cowan, 2014). Instead, whole-community metagenome (WCM) sequencing has been demonstrably the most successful method for exploring phage communities across environmental niches (Hayes et al., 2017). Metagenomic sequencing of gut microbiome free-phages relies on enrichment of VLPs, prior to nucleic acid extraction, introducing the previously discussed biases. Protocols typically use size fractionation of free phages selecting for < 200 nm, with particular focus on DNA phages (Kleiner et al., 2015). A review of the steps involved in producing phage metagenomes, including the biases associated with each step, has been reviewed by Hayes et al. (2017).

The first use of viral metagenomics (viromics) on the human gut described phages found in human faeces, finding evidence for the dominance of DNA phages (Breitbart et al., 2003). Since then, many studies have aimed to identify phages from gut WCMs (Ogilvie et al., 2013; Waller et al., 2014). While metagenomes are expected to be dominated by bacterial DNA, it has been shown that up to 17% of gut microbial DNA is predicted to be of phage origin (Ogilvie et al., 2013). The phage sequences recovered are likely to be predominantly prophages, given the abundance of bacterial DNA in gut metagenomes (Williamson et al., 2008; Stern et al., 2012). This is not a limitation, as the gut phageome of humans and mice is dominated by integrated prophages, carried by the majority of intestinal bacteria (Waller et al., 2014; Kim and Bae, 2018). Nonetheless, analysing both the viral metagenome (virome) and WCMs from faecal samples is essential to appreciate the total phage community occupying the gut niche. Furthermore, it is particularly important for deciphering the presence of a core gut phageome.

Phage populations recovered through viromics do not necessarily reflect the true diversity of the gut phageome; larger phage genomes are often only assembled for the most abundant viral genotypes present within a sample, and many lowly abundant viral species are likely to be lost in purification steps during sample preparation or biased against by the sequencing protocol. In addition, the phylogenetic binning strategy for reconstructing short reads in shotgun metagenomes may result in the loss of closely related phage contigs due to high nucleotide sequence similarity. Genome assembly and binning is complicated by mosaicism within phage genomes, a common feature of prophages (Lima-Mendez et al., 2011), which are of particular interest in the gut phageome (Waller et al., 2014; Kim and Bae, 2018). Given this, and the costs associated with achieving high sequencing depths from faecal samples, many contigs represent only fragments of the true sequenced gut phage genomes (Shkoporov and Hill, 2019). To mitigate issues with the accuracy of viral population detection, the research community has defined thresholds of contig length and read coverage to balance sensitivity with false-discovery rate (Roux et al., 2017). This includes considering phage populations as “detected” only when virome reads map at ≥ 90% nucleotide identity to non-redundant contigs ≥ 10 kb over ≥ 75% of their length. Subsequent false-discovery rates have been shown to remain low at 0.2% using these thresholds (Roux et al., 2017). Further, software tools such as CheckV (Nayfach et al., 2020) can be integrated into viromics pipelines to estimate the confidence in assembled phage genomes for downstream analyses (**Table 3**). A confidence score of > 90% completeness indicates high quality contigs, important for distinguishing short contigs as near-complete phage genomes. CheckV can also be used to identify integrated prophages through the detection of flanking host genes (Nayfach et al., 2020).

**Table 3:** Bioinformatic tools used to study the gut phageome, including techniques, research questions they may address and name of the software.

Technique	Research questions	Tools	Examples in publication
Annotate genomes	Do the phage genomes encode auxiliary metabolic genes (AMGs)?	Prodigal (Hyatt et al., 2010) or PHANOTATE (McNair et al., 2019) to predict coding sequences.	
	Do the phage genomes encode viral taxon-specific genes?	Prokka (Seemann, 2014) or eggNOG (Powell et al., 2012), to functionally annotate draft genomes.	
	Are the phages temperate or virulent?	Hmmscan (Eddy, 2011), to align protein sequences to databases (e.g. pVOGs (Grazziotin et al., 2016) and Pfam/vFam (Skewes-Cox et al., 2014; El-Gebali et al., 2019) and Genecatalog (Li et al., 2014).	(Clooney et al., 2019)
	Is there a global phage gene pool?	Phyre2 (Kelley et al., 2015), to find proteins with structural homology .	
		DRAM-v to predict putative AMGs associated with metabolism (Shaffer et al., 2020).	
Phage prediction	Are the genomes assembled phage genomes?	De-novo prediction of phages using tools such as VirFinder/ DeepVirFinder (Ren et al. 2017; Ren et al., 2002) and VirSorter2 (Guo et al., 2021)	
		Confirmation of viral genome completeness with CheckV (Nayfach et al., 2020) and IMG/VR v3 (Roux et al., 2021).	
	How diverse are the phages?	Alignment to hallmark taxon-specific marker genes.	(Waller et al., 2014)
	Are any known viral taxa predicted?	BLAST (Camacho et al., 2009), PhymmBL (Brady and Salzberg, 2009) and k-mer approaches (Trifonov and Rabadan, 2010), for taxonomic assignment.	(Vazquez-Castellanos et al., 2014)
		Comparison to gut viral databases, e.g. Gut Virome Database (GVD) (Gregory et al., 2020).	
	How similar are they to phages in other ecosystems?	Single protein phylogenies with alignment of core phage genes, e.g. using MAFFT (Katoh and Standley, 2013).	
		De-novo classification with vConTACT2 (Zablocki et al., 2019).	
Phage-host relationships	Which gut hosts do the phages infect?	Genetic homology of integration sites and CRISPR spacer sequences between phage and host.	(Stern et al., 2012)
	Is phage predation species or strain specific?	Phage genome signature-based recovery.	(Ogilvie et al., 2013)
		Identification of integrated prophage.	
		Abundance profiles.	(Dutilh et al., 2014)
		De-novo relationships with WIsH (Galiez et al., 2017)	

As a consequence of the above limitations, and the exclusion of single-stranded DNA (ssDNA) and RNA phages whose true prevalence in the gut phageome is unknown, analysis of phage diversity through metagenomics often underestimates absolute viral richness. Further, inferences on phage diversity yield only a snapshot of the sampled phage community at a given time point. The temporal dynamics of the gut microbiome can only be understood by sampling multiple time points, which adds logistical challenges of recalling participants, costs, and is both more lab- and bioinformatically-intensive due to the size of the datasets assembled and analysed. Nonetheless, there are a few examples of longitudinal studies of the gut phageome, which are discussed in section *Development of the Phageome* (Minot et al., 2013; Shkoporov and Hill, 2019).

There are a number of tools used to describe and interrogate environmental phageomes through metagenomics, reviewed in **Table 3**. Many of these tools are alignment-based, requiring sequence homology between genes and genomes for annotation, phage classification and the inference of phage-host relationships. Historically, due to the underrepresentation of viruses in reference databases, in conjunction with a high proportion of novel phages sequenced from the gut (Shkoporov et al., 2018b), alignment-based approaches were limited. It has been reported that > 75% of reads from gut viromes did not align with known viral genomes (Aggarwala et al., 2017). In another study, < 2% of the viral genomes assembled from human faeces could be assigned to known taxa (Shkoporov et al., 2018b). Additionally, both RNA (Callanan et al., 2020) and non-tailed (Kauffman et al., 2018) phages are significantly underrepresented in phage databases. Therefore, alternative tools that do not rely on similarity to databases have been proposed to circumvent these limitations (Reyes et al., 2012), but have yet to see widespread implementation. Recently, great advances have been made in assembling comprehensive databases for the detection of viral populations from gut microbiome studies, e.g. Gut Virome Database (Gregory et al., 2020). A combination of this increased viral representation in databases, software advances, and the development of novel bioinformatic tools (**Table 3**), is essential for continuing to improve the proportion of true phageomes captured by viromic analysis and understanding their functions.

Another limitation associated with assembled viral genomes is the lack of information on viral activity (Stern et al., 2012). Sequenced phages may be quiescent and thus less metabolically important relative to the active phage community. Further, the verification of host range, gene function, and replication strategy characterisation requires the cultivation of phage isolates. The activity of phages can, however, be resolved through a combination of ‘omic strategies. In a similar approach to sequencing the DNA metagenome, other meta-’omic strategies have been adopted to study the gut phageome at the RNA, protein and metabolite-levels.

### Other ‘Omics

In metatranscriptomics, the sequencing and *de-novo* assembly of RNA contigs is carried out in a similar way to metagenomics, reviewed for environmental samples in (Lim et al., 2013). Notably, for recovering RNA phages from the gut, there are inherent difficulties facing the collection, storage and assembly of RNA libraries from human samples, which have been reviewed by Bikel et al. (2015). This could be why metatranscriptomics has had limited usage in the study of the gut phageome. In addition, tools developed for the prediction of DNA phage contigs from metagenomes are not optimised for the prediction of RNA phages, due to the scarcity of known RNA phages and the vast diversity in their genomes (Duffy et al., 2008). Subsequently, RNA phages have rarely been identified in the gut; the RNA viruses that have been identified were derived from plants consumed in the diet (Zhang et al., 2005; Lim et al., 2015). A novel alternative approach to recovering RNA phages, based on homology to known core single-stranded RNA (ssRNA) phage genes, has been implemented on metatranscriptomes generated from activated sludge and aquatic environments (Callanan et al., 2020). Future studies should aim to use this method to recover RNA phages from the gut, given recent findings of their abundance and activity in other ecosystems (Callanan et al., 2018; Starr et al., 2019).

A second use of metatranscriptomics comes with its combined implementation alongside metagenomics. By mapping metatranscriptome reads to assembled phage genomes, gene transcription can be estimated as a proxy for phage activity to discern active from dormant prophages. Further, the most actively transcribed genes can be determined for the functional analysis of the phageome. When this combined approach is applied to samples over a time-course, the dynamics and persistence of phage infection can be determined, as shown for marine ecosystems (Sieradzki et al., 2019). To the best of our knowledge, metatranscriptomics has had limited implementation on the gut phageome, but future efforts should enable the investigation of active functional genes, including auxiliary metabolic genes, and discern the proportion of the phageome that is active within the gut microbiome in diseased and healthy states. This could uncover further evidence for implicating the phageome in disease.

Metaproteomics captures the protein complement of microbial communities and is a direct study of the end product of gene expression, used to evaluate the functional state of a community. Downstream analytical techniques such as mass spectrometry and nuclear magnetic resonance can be implemented to characterize isolated proteins. Modern advances in these techniques have improved the throughput and accuracy of this approach for its application on the gut microbiome (Arnold et al., 2016). Metaproteomics has been used to provide further support for the dominance of temperate phages in the gut, from an abundance of virally-encoded proteins associated with lysogenic replication strategies (Ogilvie et al., 2013). Additionally, metaproteomics has revealed phage proteins that were not identified through genome-based alignment approaches, indicating the advantage of a complementary multi-’omic strategy (Ogilvie et al., 2013). Other applications of proteomics on the gut phageome are very rare in the literature, but have been proposed to uncover the functional potential of phages (Shkoporov et al., 2019). Like metatranscriptomics, future metaproteomic analysis should enable inferences on the relative functional state of the phageome in the gut through comparative and longitudinal studies.

Metabolomics specifically targets the metabolites present within cells, thus including those involved in lysogenic conversion. In gut microbiome research, metabolomics has been used to evaluate the metabolic state of the gut microbiome as a result of phage predation (Hsu et al., 2019). This approach facilitates the direct monitoring of phage function in the gut, moving beyond the correlation of phage populations with those of their host. Future use of metabolomics could be applied to explore the role phages play in microbial community function, interactions with the human host and differences in phage-mediated community function between healthy and diseased states (see section *Role of Bacteriophages in Human Disease*).

The methods used to identify and characterize phages have come a long way since phages were first visualised in 1940 (**Figure 1**). Early culture-dependent methods isolated intestinal phages from faeces for the first time, microscopy enabled the morphotypes and abundances of gut phages to be realised, and recent ‘omic advances have facilitated the study of phageome diversity and function.

There are undoubtedly numerous additional challenges that face the study of the phageome, as compared to the bacterial microbiome. While we have provided many limitations of the existing methods, we have underlined the potential for their future development and implementation in studying the gut phageome. Namely, the greatest challenge involves expanding the proportion of the phageome captured by ‘omic analyses. This means recovering increasingly low abundant phage populations and describing their functional potential and reality. To this aim, future advances in both culture-dependent and sequencing methods, in addition to the combination of these approaches, can be achieved in a number of ways. This begins with amending sampling biases through the direct study of gut samples, and the greater representation of sampled individuals outside of Europe and North America. Increasing sequencing depth, augmenting bioinformatic tools for recovering and annotating phage contigs, and combining ‘omic techniques will maximize our ability to characterize sampled phageomes. The simultaneous implementation of culture-dependent methods with sequencing approaches can validate *in silico* hypotheses of phage-host interactions for implicating phage populations in the overall functioning of the gut.

## Composition of the Gut Phageome

### Development of the Phageome

**Figure 3:**
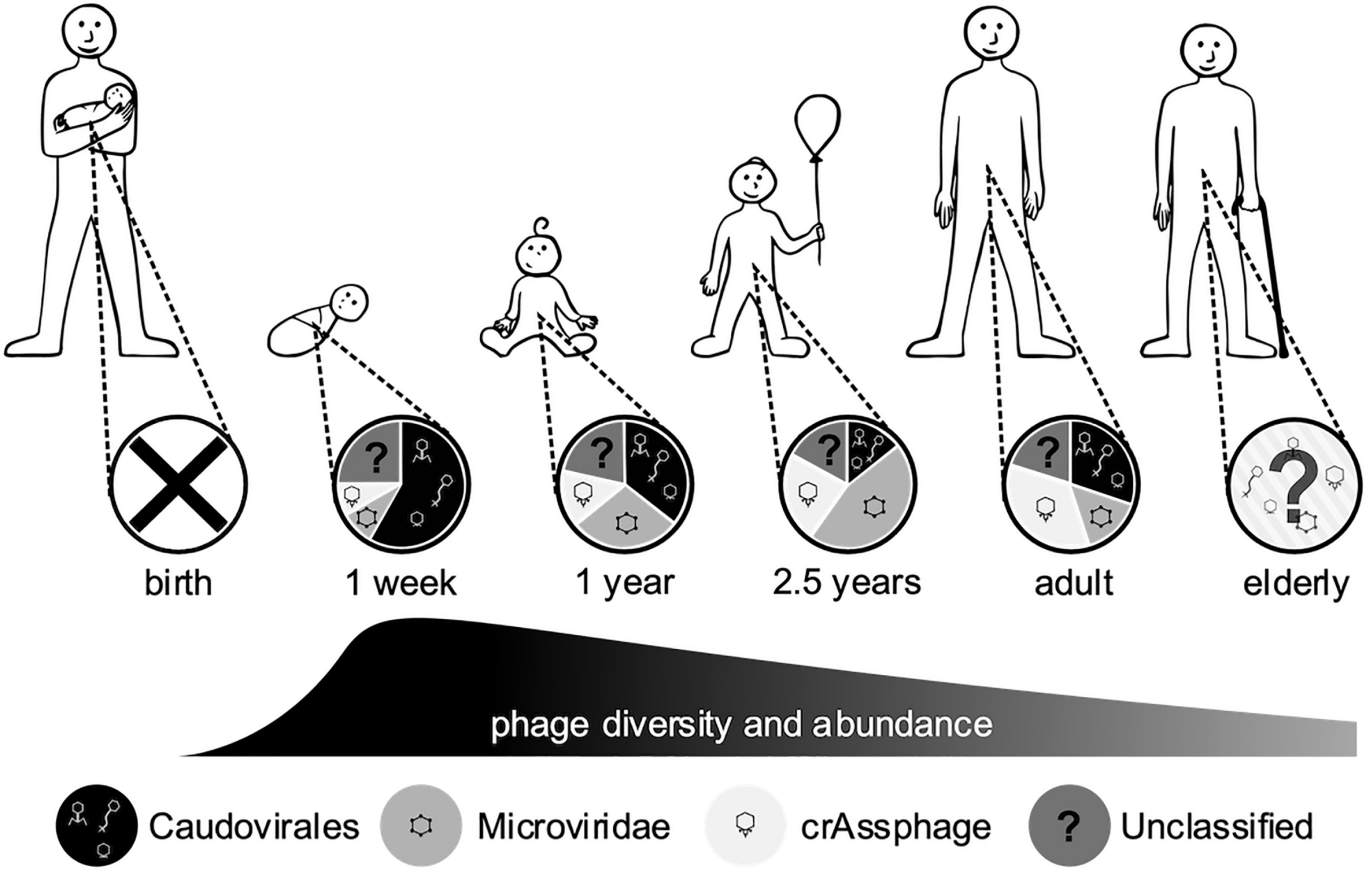
Changes in the gut phageome over the human lifetime. Pie charts represent the observed ratios of different phage groups at discrete sampling times, from birth where no endemic phages were observed to adults, while the humped line describes changes in phage diversity and abundance over time, which both peak in the weeks after birth (Breitbart et al., 2008; Reyes et al., 2010; Kim et al., 2011; Lim et al., 2015; McCann et al., 2018).

The human gut is host to a complex community of microorganisms that contribute greatly to gut function, immune responses and disease pathogenesis. The human microbiome develops from birth, is predominantly of maternal origin, and expands rapidly in response to many environmental factors (Yassour et al., 2016). The phageome follows a similar pattern to the bacterial populations, with changes in diversity occurring within the first 2 weeks of life (**Figure 3**) (Breitbart et al., 2008). Initially the phageome is of low diversity, with the sources of inoculation being unclear (Mills et al., 2013). Suggestions of the origin of this initial phageome include dietary, environmental and maternal sources (Breitbart et al., 2008), and induction of prophages from the newly colonised microbiota (Breitbart et al., 2003). As early bacterial colonisers are also derived from the maternal source, these should not be considered distinct sources but rather a multifaceted acquisition. It has been reported that vaginal delivery or caesarean section can produce distinctly different viral communities within the infant gut phageome (McCann et al., 2018). *Bifidobacterium* and their phages can be vertically inherited from the mother *via* breastmilk (Duranti et al., 2017), including prophage induced from colonizing bacteria (Liang et al., 2020) and phage that infect probiotic lactic acid bacteria abundant in breastmilk (Martin et al., 2003; Pannaraj et al., 2018). Sharon et al. (2013) found that a group of closely related *Caudovirales* bloomed, then persisted in the infant gut along with their host strains, between weeks 15-24 after birth. These *Caudovirales* initially dominate the gut phageome, then a shift to a *Microviridae*-dominated population occurs at around two and a half years old (**Figure 3**) (Lim et al., 2015). It has been postulated that the *Caudovirales* populations cannot be supported by the minimal bacterial community, leading to a phage population crash. This, in turn, allows bacterial populations to thrive without predation, shifting the microbiome towards a mature state with an increased diversity of bacteria.

In adulthood, the gut phageome has been proposed to be dominated by phages exhibiting a temperate lifestyle (Silveira and Rohwer, 2016). While this may be true, measures of the phageome are not absolute and the diversity of temperate phages may be masked by a few highly abundant lytic phages, meaning that the intricacies of the phageome have yet to be fully resolved (Sutton and Hill, 2019). The adult gut phageome shows a return to a *Caudovirales*-dominant population with a subpopulation of *Microviridae* (**Figure 3**) (Kim et al., 2011; Manrique et al., 2016), although there is a large degree of inter-individual variation (Reyes et al., 2010). Communities dominated by *Microviridae* or *CrAssphage* have also been described in some individuals (Shkoporov et al., 2019). This inter-individual diversity appears to be driven by environmental factors (Reyes et al., 2010), and is significant even between identical twin infants and families (Lim et al., 2015; Reyes et al., 2015). Some RNA phages have also been detected in the gut phageome, but in significantly lower abundance in comparison to DNA phages (Breitbart et al., 2003; Zhang et al., 2005). However, it is difficult to distinguish if this is due to a genuine reduced presence or lack of effective techniques to detect RNA phages, as discussed in section *Other ‘Omics*. Later in life, the gut microbiome decreases in diversity and shifts from a *Firmicutes-* and *Bacteroidetes*-dominated community to one dominated by obligate and facultative anaerobes (Nagpal et al., 2018). While it has yet to be investigated, the functional capacity of the phageome may also be influenced by this change in composition. Though the gut phageome seems to be highly specific from person-to-person, there has been indication of stability over time (Shkoporov et al., 2019), with sequences of the same phages detected more than 2 years apart (Minot et al., 2011; Minot et al., 2013). There is emerging evidence for a core gut phageome, Manrique et al. (2016) identified a set of 23 phages that were present in over 50% of healthy gut samples analysed, with an additional 44 phage groups present in 20-50% of individuals, suggesting a secondary set of shared phages. A recent study found that both *CrAssphage* and *Microviridae* were the two most stable members of the gut viral community, suggesting a potential role as part of a core phageome (Shkoporov et al., 2019). However, any core communities only represent a fraction of the overall population, it is almost impossible to simplify the complexity and individuality of the phageome.

### Identification of Novel Human Phages

*CrAssphage* were discovered in 2014 *via* the cross-assembly (crAss) method, providing the name *CrAssphage*. Previously undetected, they are the most abundant gut phage currently known (Dutilh et al., 2014). Through both metagenomic and culture-based studies, their host has been predicted to be *Bacteroides*, with a podoviridae-like morphology (Shkoporov et al., 2018a; Yutin et al., 2018). Interestingly, no known lysogeny genes were found in the genome of the original *CrAssphage* isolate (crAss001), yet the phage could maintain its population in culture with *Bacteroides intestinalis*, in part due to the long latent phase and very small burst size of 2.5 plaque forming units per cell. However, the true mechanism was not elucidated.

There is evidence to suggest that *CrAssphage* has long been a member of human gut phage communities, having also been found in the gut of non-human primates, such as baboons and gorillas (Edwards et al., 2019). This presents the idea that *CrAssphage* have evolved with humans and maintained their presence in the gut phageome. Furthermore, *CrAssphage* sequences have been found to persist universally across the globe. The majority of strains identified were only present in individual samples, however one strain was identified in 104 independent samples spread across multiple continents (Edwards et al., 2019). In addition, *CrAssphage* was more abundant in the gut of industrialised populations in comparison to traditional hunter-gatherer populations (Honap et al., 2020). These findings, taken together, suggest a recent human expansion event, associated with industrialisation, led to the expansion of *CrAssphage* within the human population.

The presence of *CrAssphage* in the gut appears to begin in infancy (McCann et al., 2018; Edwards et al., 2019; Shkoporov et al., 2019). It appears to be transmitted vertically, with homology found between isolates from mother and child (Siranosian et al., 2020), which also corresponds with the presence of its *Bacteroides* host (Tanaka and Nakayama, 2017). However, there is again a large degree of inter-individual variation in the presence and abundance of *CrAssphage*.

A group of megaphages (with genomes > 500 kb in length) have been identified from human faecal samples and have been tentatively predicted to infect *Prevotella* (Devoto et al., 2019). These “Lak phages’’ were detected in a wide range of samples and are exceptional, not only for the size of their genomes but also, for their use of a genetic code with an alternative stop codon, setting them apart from other phage families (Devoto et al., 2019). Lak phages are yet to be isolated, but the size of their genomes again raises the question of how many large (> 200 nm) phages are excluded by size fractionation (see section *Culture-Dependent Methods*).

Unusual, composite phages have also been identified to play a role within the gut. Bacterial strain *Enterococcus faecalis* V583 contains multiple prophage elements, two of which combine to form a lytic phage particle (Duerkop et al., 2012). One of these distinct prophages encodes the structural proteins, while the other contains necessary infection elements, and both are essential for lytic infection. The resultant phage appears to give *E. faecalis* V583 a competitive advantage over related strains when colonizing the gut (Duerkop et al., 2012) (see discussion in section *Phage-Host Population Dynamics*). While being of interest as a stand-alone case, this also has implications for future metagenomic studies when partial prophage-like sequences are found.

### Longitudinal Distribution of Phages in the Gastrointestinal Tract

The location within the gut also influences the phageome (**Figure 2**). Following the stomach, the proximal gut provides the most hostile environment for bacteria, a microaerophilic environment with a low pH and antimicrobial peptides which enriches bacteria such as Lactobacillaceae and Enterobacteriaceae. The distal gut is the opposite, an anaerobic environment, with a higher pH and reduced concentration of antimicrobial peptides, allowing bacteria such as Bacteroidaceae, Prevotellaceae and Ruminococcaceae to thrive (Donaldson et al., 2016). This longitudinal gradient causes a steady increase in bacterial load and diversity from the proximal to the distal gut, and it may be expected that the phage distribution would also follow this longitudinal axis. In primate models, it has been shown that both the relative abundance of phages and the composition of the phageome varies depending on site within the gastrointestinal tract (Zoetendal et al., 2012; Zhao et al., 2019). Furthermore, a study in pigs found a higher abundance of phages in the ileum, and hypothesised that the nutrient-limited environment may prevent bacterial phage resistance and promote pseudolysogeny, allowing phage proliferation (Looft et al., 2014). The density of phage populations also varies cross-sectionally through the mucus layers of the gut (Barr et al., 2013a), the implications of this are discussed further in section *Interactions With the Immune System*. These differences in phage communities hint at the different roles and functions of phages throughout the gastrointestinal tract, that require greater understanding to use phages in the future as markers of gut health or as treatments to improve gut function. In **Figure 2** we have displayed the varying composition of the phageome throughout the digestive tract, where the majority of the knowledge arises from faecal samples.

### Impact of Diet on the Phageome

A well-characterised influence on the gut microbiome is diet, therefore it can be assumed that diet will impact the phageome too (Singh et al., 2017). This has been observed in studies of high fat diet (HFD) in mouse models, wherein both the microbial and viral gut communities were affected (Howe et al., 2016; Schulfer et al., 2020). These changes to the phageome during a long-term HFD included an increased abundance of *Microviridae*, while *Siphoviridae* abundance decreased (Schulfer et al., 2020). As breast milk is high in fat, this may provide some insight into why previous studies have found that infant gut phageomes move towards *Microviridae*-dominated communities (see section *Development of the Phageome*) (**Figure 3**). The change of diet in infancy, to weaning and solid foods was shown to alter gut phage composition and diversity in pandas (Guo et al., 2020), suggesting the change from infant to adult phageome is in part influenced by dietary factors. Malnutrition in infancy was also found to affect the gut phageome (Reyes et al., 2015). The microbiome and phageome developed sequentially, but the phageome was disrupted in malnourished infants (Reyes et al., 2015). Phage populations could not be recovered, despite the use of dietary supplements, suggesting that infancy is a key time in the development of the gut phageome (Reyes et al., 2015). Further studies have identified that the phageome is not only distinct in malnourished children, but in fact these phages may play a pathophysiological role (Mirzaei et al., 2020). *In vitro* experiments indicated phages from a stunted child caused microbiota shifts to a disease associated state in an age-related manner (Mirzaei et al., 2020). The study suggested there is a time window where the phageome and microbiome of children less than 23 months is amenable, before the malnutrition-associated community becomes fixed. As malnutrition and stunting of growth is a major cause of mortality in infants, this finding is of great importance.

Once the phageome is established it is less influenced by diet, but increasing fat does seem to have an impact. Increased fat leads similar shifts in the gut phageome, however there was still great inter-individual variation (Minot et al., 2011). This suggests that the phageome is not acquired through diet, but that the diet shapes existing communities (Minot et al., 2011). In addition, consumption of various foods can influence prophage induction in the resident gut microbiota (Kennedy et al., 1984; Jofre et al., 1995; Méndez et al., 2004).

Numerous studies have identified food products that are a rich source of bacteriophages, yet there is little evidence if or how they influence the phageome. Coliphage can be found in a variety of food sources, and are used as an indicator of faecal contamination (Kennedy et al., 1984; Kennedy et al., 1986; Jofre et al., 1995; Méndez et al., 2004). Gut viruses include eukaryotic and plant viruses that arrive in the gut *via* the oral route (Liu et al., 2013), which have been demonstrated to make up a high proportion of RNA viruses detected in the gut (Zhang et al., 2005). There is also evidence of bacteriophages surviving food decontamination processes, such as fermented foods; including soy beans (Chukeatirote et al., 2018), sauerkraut (Yoon et al., 2002; Lu et al., 2003), cheese (McIntyre et al., 1991; Quiberoni et al., 2006), yoghurt (Ma et al., 2014; Ma et al., 2015), fermented vegetables (Lu et al., 2003; Kleppen et al., 2012), fermented meats (Trevors et al., 1984), drinking water (Armon et al., 1997) and heat-stable phages surviving dairy product pasteurisation (Suarez et al., 2002; Murphy et al., 2014). Furthermore, in recent years, there has been a rise in the consumption of unpasteurised milk, unfortunately linked to a number of bacterial diseases (Lucey, 2015). Unpasteurised milk has been demonstrated to be an abundant source of new bacteriophages, containing up to 10^4^ bacteriophages per mL of product (Marco et al., 2012). To date, no studies have examined the effect of dietary phage intake to understand the impact of normal dietary intake on the phageome and wider microbiome.

Bacteriophages are currently being used to make food safer and have been used as indicators for the virological safety of food (Allwood et al., 2004), as alternatives to antibiotics in animal husbandry (Cheng et al., 2014; Gigante and Atterbury, 2019), in food decontamination (Moye et al., 2018), to increase yield or quality (Zhao et al., 2012), and to prevent spoiling (Sillankorva et al., 2012). **Table 4** displays a list of bacteriophage products approved for use in food manufacturing. As the use of these phage products become increasingly more common, their abundance in the human diet is likely to increase; the impact on the human gut microbiome is currently unknown. Drinking water is another potential source of phages, regardless of high quality water treatment systems and microbiologically safe drinking water, viruses survive the process (Armon et al., 1997). While large scale changes due to dietary consumption of phages appear transient, there is the potential for a variety of viruses to enter the digestive system and influence the human host.

**Table 4:** Phage products approved for use in food manufacturing. These phage products have the opportunity to enter the GI tract and impact the gut phageome.

Manufacturer	Product	Applications
Intralyx Inc. (MD, USA)	ListShield™	Targets *Listeria monocytogenes* contamination in foods and food processing facilities.
	EcoShield™	Targets Escherichia coli, and Shiga toxin producing *E. coli* in particular, including O157:H7 STEC.
	SalmoFresh PX™	Targets contamination with selected, highly pathogenic *Salmonella*-serotypes in foods and food processing facilities.
	ShigaShield™	Targets contamination with *Shigella* spp. in foods and food processing facilities.
APS Biocontrol (Dundee, UK)	Biolyse®-PB	Targets bacteria that cause soft rot on potatoes mainly, *Erwinia* spp., *Pectobacterium* spp., and *Pseudomonas* spp.
Proteon Pharmaceitocals	BAFASAL®	Targets human-pathogenic *Salmonella* spp. in poultry farming.
	BAFADOR®	*Targets Pseudomonas* spp. and *Aeromonas* spp. in commercial aquaculture
Elanco (IN, USA)	Finalyse™	Pre-slaughter hide wash applied to live cattle, targeting *E. coli* O157:H7
Micreos Food Safety, (The Hague, NL)	PhageGuard Listex™	Surface treatment targeting *Listeria monocytogenes* on a number of food products such as, meat, fish, cheese and frozen vegetables.
	PhageGuard S™	Targets *Salmonella* spp. on fresh poultry.
	PhageGuard E™	Targets *E. coli* O157 on beef carcuses.
OmniLytics (UT, USA)	Agriphage™	Targets *Xanthomonas campestris. Xanthomonas vesicatoria* and *Pseudomonas syringae* to prevent bacterial spots tomatoes and peppers.

The human gut is an abundant source of potential host bacteria, which should allow many dietary phages to thrive. Equally there are a number of factors that may prevent colonisation by dietary phages, including phage-specific antibodies (Mirzaei and Maurice, 2017), eluded to by a higher abundance and diversity within the phageome of patients with immune disorders (Norman et al., 2015; Perez-Brocal et al., 2015). Phage colonisation may be further hampered by various exclusion strategies by host strains (Rossmann et al., 2015) and phage competition (Reyes et al., 2013). It has been shown that orally delivered phages can survive the stomach to be detected in the faeces, while some phage treatments reduce target bacteria (Mai et al., 2015), not all phage-host systems are equal and some phages fail to proliferate (Sarker et al., 2016). With GI tract phageome studies limited in scope and number, the lack of direct supporting evidence for dietary phages to shape the phageome and microbiome does not mean that they play no role. Instead, it is another overlooked aspect of the human gut microbiome, particularly in the first two formative years.

### Impact of Medical Interventions on the Phageome

Medical procedures and treatments can have an effect on the gut phageome, including the well-documented impact of antibiotics. The effect of broad spectrum antibiotics on the gut bacterial community is non-targeted killing, leading to a dysbiotic state (Jernberg et al., 2010), which may in turn affect the phageome. In rhesus monkeys there has been an observed decrease in viral abundance and diversity following antibiotic treatment (Li et al., 2019), but no detectable change in the gut phageome was found in human studies of long-term antibiotic use (Abeles et al., 2015). It may be that disturbances in the gut viral community caused by antibiotics are rapid, and during long-term exposure the community re-adjusts and stabilises. In the short-term, prophage induction has frequently been demonstrated upon antibiotic exposure due to host DNA damage and initiation of the bacterial SOS response, in turn activating prophages and potentially increasing the likelihood of horizontal gene transfer (Maiques et al., 2006; Allen et al., 2011; Lin and Lin, 2019), further discussed in section *Virus-Mediated Genetic Exchange*. This has consequences for subsequent antibiotic usage, if a reservoir of phage-encoded antibiotic resistance genes is established in the gut (Modi et al., 2013; Abeles et al., 2015).

Faecal microbiota transplant (FMT) is the transfer of faecal material from a healthy donor to an individual with gastrointestinal disease with the intention to restore a healthy gut microbiome. It has been shown that the phage component is of high importance in FMT, able to improve efficacy (Bojanova and Bordenstein, 2016; Zuo et al., 2018) and remains effective without the bacterial fraction of FMT (Ott et al., 2017). The mechanism behind this has not yet been established; it may be due to an alteration of the patient’s own phageome or the predatory action of the donated phages. A number of studies have looked at the potential of faecal virome transplants (FVT) from one individual to another, which could assist recovery after antibiotic-induced dysbiosis (Draper et al., 2019).

In contrast with the microbiome, there has been limited success in defining a core phageome. The phageome is highly specific to an individual and its resilience to change over time raises interesting questions. The phageome appears to remain relatively constant, while we know the microbiome fluctuates over time and with environmental factors. But are there as yet undetected effects on the phageome? Do phage populations remain dormant for a long time, persisting in host populations, or is their host range wide enough that they continue to encounter hosts regardless of fluctuations in bacterial populations? We know that phages are consumed in the diet, and it is assumed with the survival of other viruses, phages also survive the human gastrointestinal tract. If this is the case, are phages able to colonize the gut once they reach it? As seen with FMT, this appears to be possible as phages seem to be the key to success of this treatment. However, the stability of the phageome within an individual suggests that no such invasion and colonisation occurs. Answering this myriad of questions is hampered by the sampling method used to study the phageome: metagenome sequencing of faeces (see section *Metagenomics*). Measuring phage activity throughout the GI tract over a time course would help to elucidate fluctuations in the phageome at sites where it could impact human health and gut function. As this is still an emerging field, we hope with greater investigation the phageome will reach a level of understanding we see for the microbiome.

## Phageome, Gut Bacteria, and Human Host Interactions

Given that most phages infect and eventually lyse specific bacterial hosts, the gut bacterial community shapes the phage community, as much as it is shaped by phages. Phage-host interactions occur in a number of context-dependent dynamics throughout the gastrointestinal tract, playing roles in the cycling of nutrients, community function and transfer of genetic material. The role of the phageome is seen primarily through its influence on the function of the bacterial community, however there are further consequences of their presence in the gut, including on human immune responses, impacts on human health and gut-associated bacterial biofilms.

### Phage-Host Population Dynamics

In the human gut, the phageome is thought to affect succession and colonisation events (Lozupone et al., 2012; De Sordi et al., 2019; Shkoporov et al., 2019), it is well known that phage contribute to feedback loops and predation which influence the dominance and diversity within a microbial population. Broadly speaking, there are three main dynamics under which the phageome will be able to alter the gut microbiome: Red Queen dynamics [continual evolution of defence and counter-defence measures (**Table 5**)] (Van Valen, 1973), kill-the-winner (lysis of common genotypes, preventing dominance) (Maslov and Sneppen, 2017), and piggyback-the-winner (lysogeny allowing phage and bacteria to stably co-exist) (Weitz and Dushoff, 2007).

**Table 5:** Summary of bacteria-encoded phage defence mechanisms.

Mechanism		Infection stage disrupted	Example references
Modification of phage receptors		Phage attachment and adsorption	(Howard and Tipper, 1973; Sørensen et al., 2011)
Masking of phage receptors	with protein	Phage attachment and adsorption	*Staphylococcus aureus* (Nordström and Forsgren, 1974)
			*Escherichia coli*, preventing superinfection (Pedruzzi et al., 1998)
	with polysaccharides		*Escherichia coli* (Stummeyer et al., 2006)
Phase variation of phage receptors		Phage attachment and adsorption	*Bordetella pertussis* (Uhl and Miller, 1996)
Production of competitive inhibitors to phage receptors		Phage attachment and adsorption	*Escherichia coli* (Destoumieux-Garzón et al., 2005)
Superinfection exclusion (Sie) systems, often encoded for by prophage.		Block phage DNA entry into cell.	*Escherichia coli* (Lu and Henning, 1994)
			*Salmonella* sp. (Hofer et al., 1995)
			*Lactococcus lactis* (Akçelik, 1998)
			*Streptococcus thermophilus* (Sun et al., 2006)
Bacteriophage exclusion (BREX)		Prevents phage DNA replication.	*Bacillus cereus* (Goldfarb et al., 2015)
Restriction-modification systems		Degrade phage DNA.	*Staphylococcus aureus* (Sjöström et al., 1978)
		*Salmonella* (Bullas et al., 1976)
Defence island system associated with restriction–modification (DISARM)		Degrade phage DNA. Type of R-M system.	*Bacillus paralicheniformis* (Ofir et al., 2018)
CRISPR/*Cas* elements		Result in degradation of phage nucleic acids.	*Streptococcus thermophilus* (Deveau et al., 2008)
Abortive infection (Abi)		Prevent phage multiplication (replication, transcription, or translation). Result in death of infected host cell.	*Lactococcus lactis* (Chopin et al., 2005)
			*Escherichia coli* (Molineux, 1991)
Toxin-antitoxin		Leads to abortive infection.	*Pectobacterium atrosepticum* (Fineran et al., 2009)
			*Escherichia coli* (Pecota and Wood, 1996)

**Figure 4:**
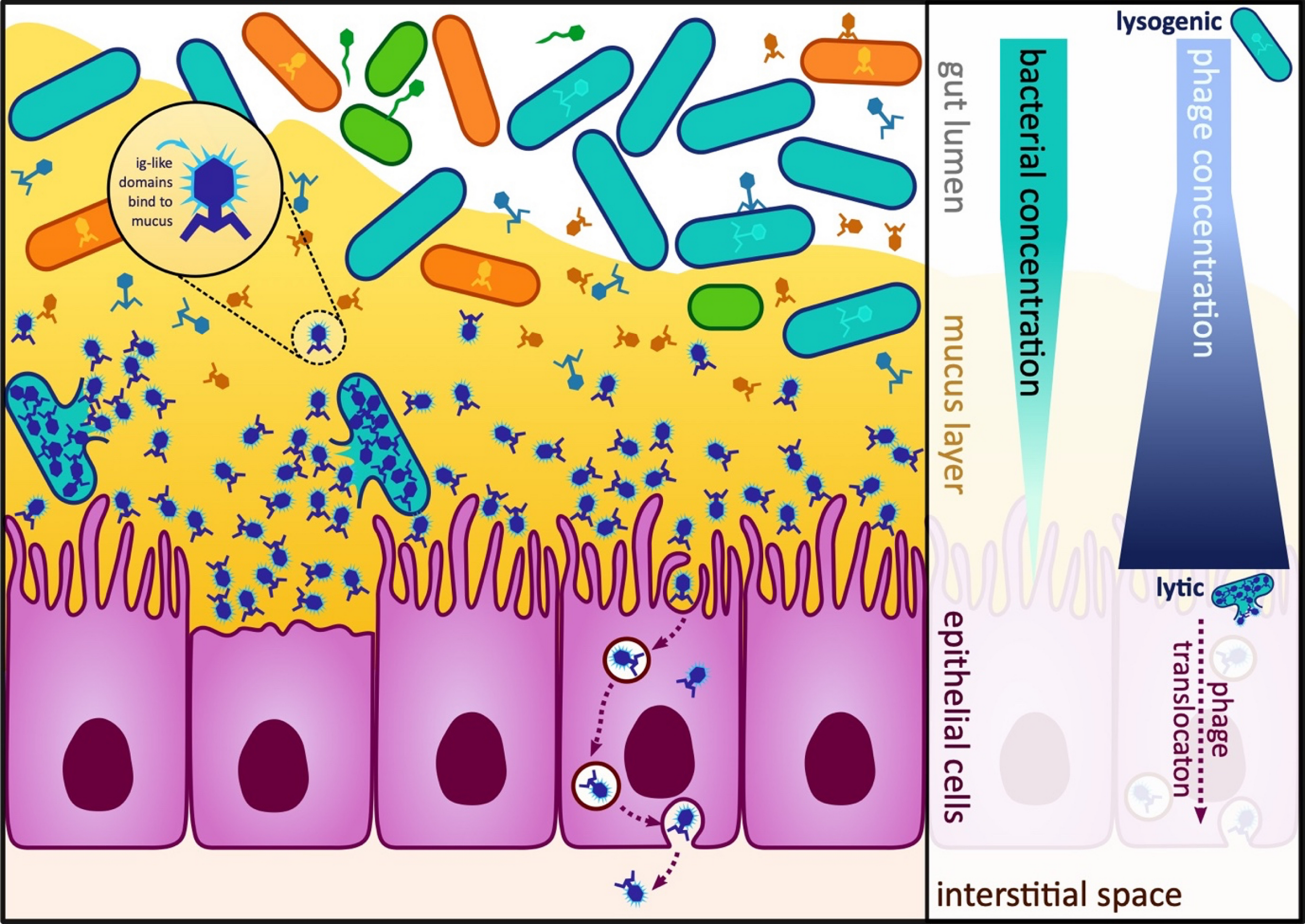
Diagram of the gut and bacterial and phage concentrations in the lumen, mucosa and epithelial cells. In the human gut, multiple different host-prey dynamics are theorised to occur dependent on the proximity to the gut mucosa. At the top of the mucosa, viruses take a lysogenic strategy or “piggyback-the-winner” as hosts are abundant here. Deeper within the mucosa, the viruses switch to a lytic or “kill-the-winner” strategy as at this point the bacteria are less abundant (Silveira and Rohwer, 2016). (Right) Some phages encode Ig-like domains that allow them to bind to the mucosa and evade the immune system, (Left) Phages can undergo transcytosis and be engulfed and transported through epithelial cells.

On the mucosal surfaces of the gut, the bacterial population is spatially structured through the surrounding mucus. The lifestyle of phages is dependent on host availability: less available hosts, lytic predation takes place, and otherwise, lysogeny is the key mode of phage lifestyle (**Figure 4**). It is theorised this kill-the-winner strategy promotes elimination of potential pathogens deep in mucus layers, while a lysogenic piggyback-the-winner strategy provides an advantage for bacterial commensals against niche invasion (Silveira and Rohwer, 2016).

As a consequence of these changes and alterations in phage dynamics and prevalence in the human gut, it is possible that no specific genotypes maintain dominance, but are constantly superseded by functionally equivalent strains, maintaining stable metabolic potential and taxonomic diversity. This functional redundancy is known to be favoured in the bacterial microbiome (Ley et al., 2006), allowing more resilience and less impact of dysbiotic perturbations (Vieira-Silva et al., 2016).

### Virus-Mediated Genetic Exchange

Bacteriophages are able to influence evolution and diversification of bacterial communities through horizontal gene transfer (Wommack and Colwell, 2000; Koskella and Brockhurst, 2014). As previously discussed, lysogenic phage and prophage dominate the human gut (Breitbart et al., 2003; Reyes et al., 2010; Minot et al., 2011). Lysogenic conversion of bacterial phenotype has been shown to contribute to 5% of the conserved microbiome function in the human gut, indicating these prophage exert a strong influence over the microbial function in this environment (Qin et al., 2010). The effects of genetic exchange are well-studied in pathogenic species, but have also been demonstrated in human gut commensals such as *Lactobacillus gasseri* (Baugher et al., 2014).

Further to the impact on the bacterial hosts, viral genetic exchange from the gut microbiome has wider implications for the mammalian host, with viral integrases being shown to mediate chromosomal integration in human cells (Groth et al., 2000). The larger impact of this has yet to be explored.

Due to the global threat of rising antimicrobial resistance, the contribution of viral genetic exchange has been studied in depth (Balcazar, 2014). It has been theorised that bacteriophage could be the missing link, transferring antimicrobial resistance genes (ARGs) from the environment into the human microbiome, as well as between bacteria within the microbiome (Muniesa et al., 2013; Quiros et al., 2014). Phages may contribute more to the dissemination of these genes compared to plasmids and chromosomal elements, both over time-scale and wider spaces (Debroas and Siguret, 2019). This will be especially important in the densely populated human gut, where numerous transduction events potentially occur at any given moment. In a mouse study, after exposure to antibiotics, there was an enrichment of phage-encoded genes associated with antibiotic resistance and/or antibiotic mitigation in faecal samples (Modi et al., 2013). This was not only to the applied antibiotic, but also to other classes. Critically, these phage-encoded genes were shown to be functional through transfecting antibiotic naïve mice (Modi et al., 2013). On a cautionary note, while these phages were able to transfer antibiotic resistance between mice, the true rate of antibiotic resistance encoded by phages is much lower than predicted by bioinformatic methods (Enault et al., 2017). Despite this, phages can facilitate the release and exchange of plasmid-encoded ARGs when they lyse their hosts.

### Cycling Key Nutrients

Nutrient cycling by phages is well-characterised in environmental systems, such as marine and soil ecosystems. The “viral shunt” explains how carbon and other intracellular nutrients are released upon lysis of host cells into the extracellular milieu. (Wilhelm and Suttle, 1999). The human gut has significantly more available nutrients compared to marine and soil systems, leading to the extremely high cell densities, resulting in more competition for available nutrients. Examples of key nutrients which are limited for the gut microbiota are iron and vitamin B_12_ (cobalamin) (Deriu et al., 2013; Degnan et al., 2014). It has been proposed that gut viruses play a role in alleviating this competition, limiting growth, regulating community stability and nutrient cycling, as observed in other systems (Lozupone et al., 2012). Due to the high degree of interindividual gut community variation, it is difficult to pinpoint how the phageome alters nutrients in the gut or is itself altered by incoming nutrients from the human host diet (see section *Impact of Diet on the Phageome*) (Minot et al., 2011; David et al., 2014).

### Interactions With the Immune System

While phage interactions with their bacterial hosts are of vital importance, phage interactions with the human host are also paramount. There is evidence that the phageome is able to influence both the innate and adaptive system, playing a defensive role at the gut mucosa (Barr et al., 2013b; Barr et al., 2015). It has been shown that immune stimulation can be caused by a number of phage proteins (Majewska et al., 2015; Miernikiewicz et al., 2016), and possibly cause a low-grade inflammation in the intestines, without any clinical symptoms (Norman et al., 2014). However, the correlation of phage in FMT with positive outcomes in inflammatory bowel disease (Gogokhia et al., 2019) [see section *Inflammatory Bowel Disease (IBD)*] suggests a more complex relationship between the phageome and individual human health.

Phage proteins, not involved in the attachment to the bacterial host, have the ability to instead attach to the gut mucosa. These adhesins have been identified on the capsid, collar whiskers and tail shaft of the phages, and possess immunoglobulin-like (Ig-like) domains (Fraser et al., 2007; Barr et al., 2013a). Along with these Ig-like domains, regions of hypervariability in the virus tail fibres help to mask phages from the immune system (Doulatov et al., 2004; Minot et al., 2012). The Ig-like domains additionally function to bind the phages to the mucus, allowing phages to form a defensive barrier over mucosal membranes (**Figure 4**). This barrier is evidence of a symbiotic coevolution between phages and the human host, by enabling phages to come into contact with bacterial prey and regulating the bacterial populations at this surface, benefiting human health (Barr et al., 2013a). Bacteriophages have been shown to be actively transported across the gut epithelium, by transcytosis to variable degrees (Bruttin and Brussow, 2005; Gorski et al., 2006; Nguyen et al., 2017) (**Figure 4**), meaning there is more opportunity for interaction with immune cells outside of the gut.

It is clear from the summative research conducted that phages, in addition to affecting their bacterial hosts, impact the health of the human or mammalian hosts in which they reside. This could have an important impact for numerous diseases, as discussed later in section, *Role of Bacteriophages in Human Disease*.

### Phage Behaviour Within a Biofilm Environment

There is evidence to suggest that the majority of microbial life within the gastrointestinal tract exists as a biofilm, a distinct lifestyle to planktonic bacteria (Costerton et al., 1987; Macfarlane and Dillon, 2007; Hussain et al., 2020). As the bacterial host behaves significantly differently within a biofilm-dominated environment, it is important to consider how this will impact phage behaviour which is predominantly investigated in planktonic *in vitro* settings.

The biofilm within the gut lumen performs multiple functions, but one of the most important is acting as a physical barrier to the mucosa. Commensal bacteria are able to form a protective coating over the mucosa which will prevent pathogenic bacteria penetrating to the epithelial cell layer (Iacob et al., 2019). However, pathogenic bacteria may invade the biofilm, lay dormant and benefit from the surrounding cells by acquiring ARGs and evading host defences. This may then lead to a disease state when the cells exit dormancy (Tytgat et al., 2019).

The self-produced extracellular matrix (ECM) of a biofilm is recognised as a protective coating of cells, preventing diffusion of small molecules through the bacterial population (Flemming and Wingender, 2010). Numerous phages have been shown to encode depolymerases which are able to degrade ECM, allowing them to make contact with host cell receptors (Hughes et al., 1998). As these are widespread, it appears to indicate that the ECM is a major barrier to phage infection (Pires et al., 2016).

However, phages are also able to promote the formation of a thicker biofilm, which as previously stated can be associated with disease. Quorum sensing, a communication system within biofilms, is able to induce temperate phages. These phages are then able to contribute to HGT within the biofilm, as well as bacterial host evolution and adaptation through insertional activation of genes (Rossmann et al., 2015; Davies et al., 2016). Phages can also provide physical additions to the ECM, through both the presence of phage particles, and released cellular contents after lysis, particularly eDNA (Rice et al., 2009; Schuch and Fischetti, 2009; Carrolo et al., 2010; Wang et al., 2010; Shen et al., 2018).

The presence of biofilm in the gut lumen is a double-edged sword; it protects the human host but has the potential to harbour pathogens and enhance disease, as seen in inflammatory bowel disease (Swidsinski et al., 2005). Equally, phages can both promote or disrupt this biofilm state. Therefore it is difficult to say what the wider impact of phage populations are in this case. These interactions should be considered before the application of phage therapy, where there may be unintended consequences of disrupting “healthy” biofilms or enhancing “diseased” biofilms.

Understanding how a single phage interacts with its infection host is quite different to how a population of phage interacts with not only bacteria but human cells, fungi and archaea which may dwell in the gut. It has been shown *in vitro* that a bacteriophage can seemingly impact on *Candida albicans*, a dimorphic fungus that can be found in the gut (Nazik et al., 2017). In this section, we explained how phages may prime the immune system, but to our knowledge there is no investigation into interactions with other non-bacterial cells. As gut communities become more widely studied, this may soon be elucidated. Having a controlled system is beneficial as it allows determination of these minute interactions, but it is no use if it does not reflect the *in vivo* situation. One *in vitro* system which could be utilised complex interaction studies, is a gut fermentation model seeded with faecal samples, currently used in the study of phage therapy for *C. difficile* (Nale et al., 2018). By expanding to a more “wide-angle lens” of phage interactions, would open the possibility to discover important off-target effects in microbial and human cells.

## Role of Bacteriophages in Human Disease

In recent years, our understanding of the intestinal microbiome has increased to encompass the ways in which it impacts on human health and disease. The majority of this research has focussed on the bacterial populations, yet the phageome is also expected to have a distinct role in shaping the gut environment (**Table 6**). Even plant viruses which enter through the diet (section *Impact on Diet on the Phageome*), such as *Pepper mild mottle virus*, elicit an immune response in humans. This suggests that viruses’ effects are not limited to infective host range (Colson et al., 2010).

**Table 6:** Summary of changes to the gut phageome in different disease states.

Disease	Bacteriophage Richness	Bacteriophage Diversity	Reference
Crohn’s disease	+	+	(Lepage et al., 2008)
	+	+	(Perez-Brocal et al., 2015)
		+	(Norman et al., 2015)
	–		(Fernandes et al., 2019)
	+		(Wagner et al., 2013)
Ulcerative colitis	+		(Lepage et al., 2008)
	+	–	(Zuo et al., 2019)
	+		(Gogokhia et al., 2019)
Type 1 diabetes		–	(Zhao et al., 2017)
		+	(Tetz et al., 2019)
Type 2 diabetes	+		(Ma et al., 2018)
Human Immuno Virus	+		(Li et al., 2012)
Cardiovascular disease	+/-		(Jie et al., 2017)
	+		(Han et al., 2018)

Phages can have both direct and indirect impacts upon the gut environment, leading to systemic consequences for human health. Phage predation of gut bacteria, particularly those that have protective roles in human health, can lead to dysbiosis and disease.

For example, the diseased gut environment can be inflammatory, leading to prophage induction in *Salmonella*, causing bacterial lysis and initiating gut dysbiosis (Diard et al., 2017). As previously discussed in section *Interactions With the Immune System*, phages can also control populations of invasive bacteria in the gut and have a role maintaining the intestinal barrier function (Barr et al., 2013a). Conversely, gut phages can also directly increase intestinal permeability, leading to translocation of bacteria and bacterial products from the gut into the bloodstream, which contributes to a chronic inflammatory response (Tetz et al., 2017).

### Inflammatory Bowel Disease (IBD)

Crohn’s disease (CD) and ulcerative colitis (UC) are the two major types of IBD. CD is characterised by chronic inflammation throughout the gastrointestinal tract, most commonly impacting the ileum and colon, whereas UC involves inflammation and ulceration limited specifically to the colon and rectum. CD can affect the entirety of the intestinal wall, leading to the development of complications such as abscesses, fistulas and strictures, whereas UC affects the inner intestinal lining to cause crypt abscesses and cryptitis (Khor et al., 2011). Understanding of IBD pathology has shown that genetic predisposition interacts with the gut microbiota and environmental cues, leading to the development of disease. On the surface IBD shows no obvious direct links to the gut phageome, however we are only beginning to understand how phages are an intrinsic part of the gut community and how they are linked to IBD.

Individuals with CD may show an altered phageome compared to healthy control subjects. Epifluorescent microscopy of biopsies from both ulcerated and non-ulcerated tissues, from CD and control patients, showed over 10-fold higher VLPs in individuals with CD, than in the control biopsies (Lepage et al., 2008). These VLPs were consistent with *Siphoviridae*, *Myoviridae* and *Podoviridae* morphotypes, and were detected more frequently in non-ulcerated mucosa, when compared to ulcerated tissues from the same individual (Lepage et al., 2008). The diversity of the gut phageome is higher in CD patients compared to healthy controls, and again higher in new-onset compared to active CD patients (Norman et al., 2015; Perez-Brocal et al., 2015). In contrast to these studies, (Clooney et al. (2019) found little change in diversity of phage populations. However, they did observe a reduction in health-associated virulent phages, with an increase in unique temperate phage populations which became predominantly lytic rather than lysogenic (Clooney et al., 2019). Within UC patients, there were subtle changes in the phageome during a disease flare up or remission state. The authors highlighted again that it is difficult to elucidate if these changes are initiated by the bacterial or phage populations. Regardless, these viral markers may be involved in mediating an inflammatory response, be induced in response to the CD patients intestinal inflammation, or potentially both.

In addition to a general increase in the abundance of phages seen in patients with CD, the emergence of dominant phage families has been studied in the gut viral community. *Caudovirales* are the most dominant phage family present in CD patient samples (Wagner et al., 2013; Norman et al., 2015; Fernandes et al., 2019). An inverse correlation was observed between *Caudovirales* richness and diversity compared to bacterial richness and diversity, indicating that the increase in *Caudovirales* is not linked to host numbers in CD (Norman et al., 2015), but rather that phage may be the driving force behind fluctuations in disease. A decreased abundance of *Microviridae* has also been reported in CD patient samples (Fernandes et al., 2019).

*Bacteroides* is commonly identified as the host of these phages, with the two most prominent phages identified infecting *Bacteroides fragilis*, a bacterium associated with healthy gut function (Kang et al., 2010; Wagner et al., 2013). As *Bacteroides* is the dominant bacterial population in CD, the role of these phages may warrant further investigation (Murphy et al., 2008; Andoh et al., 2011). As the levels of both phage and host increase, this could indicate a lysogenic relationship or blooming of the host in CD. *Faecalibacterium prausnitzii* also plays a key role in CD and mediates anti-inflammatory effects; a reduction in *F. prausnitzii* has been associated with the onset of CD (Martinez-Medina et al., 2006). Metagenomic analysis shows patients with IBD have increased numbers of *F. prausnitzii* phages (Cornuault et al., 2018), suggestive of a predatory phage function. Experimental administration of a phage cocktail against pathogenic Enterobacteriaceae, Streptococcaceae and Staphylococcaeceae, led to an off-target decrease in gut *Faecalibacterium* abundance, considered indicative of inflammation and a change in gut permeability (Tetz et al., 2017). Potentially, these off-target phage effects evidence that our knowledge of phage-host ranges may not be as narrow as we expect, and could have a downstream impact on symbiotic gut bacteria association with healthy gut function (Tetz et al., 2017). It is often assumed that phage therapy minimally disrupts a patient’s microbiome, however it may lead to unforeseen downstream effects in the gut.

In parallel to CD, the gut mucosa of individuals with UC contains an increased abundance of *Caudovirales* compared to healthy subjects, though unlike in CD, a lower phage diversity is seen (Zuo et al., 2019). Mouse studies associated this with intestinal inflammation and a more severe disease presentation (Gogokhia et al., 2019). A specific increase in *Escherichia*, *Streptococcus* and *Enterobacteria* phages is seen in UC patients (Zuo et al., 2019). Previous research has found the abundance of these bacteria to be lower in those with UC when compared to healthy controls, suggesting predatory phage or prophage induction, rather than the phageome mirroring host bacteria abundance (Michail et al., 2012). Furthermore, phage functions associated with host bacterial fitness and pathogenicity were higher in the UC gut mucosa (Zuo et al., 2019). In mouse studies, those with colitis have more phages which are not associated with their bacterial hosts, suggesting a state of intestinal dysbiosis (Duerkop et al., 2018). In a number of studies with UC patients, inflammation of gut mucosa translated to an increase in viruses, particularly *Caudovirales* phages, when compared with the non-inflamed mucosa, indicating that *Caudovirales* could play a direct role in inducing inflammation in the gut (Lepage et al., 2008; Zuo et al., 2019). Enrichment of gut phages appears to be specific to the UC disease state, as healthy controls showed an enrichment of eukaryotic viruses (Zuo et al., 2019).

Gogokhia et al. (2019) found that intestinal *Caudovirales* correlated with the success of FMTs in humans with UC. UC patients that responded well to FMT had a lower abundance of *Caudovirales* before FMT than after treatment, suggesting that the intestinal phage community could worsen disease (Gogokhia et al., 2019). This has implications for the potential use of FMT therapy and clinicians should consider the impact of phages on patient outcomes.

Comparing phage populations of CD and UC patients shows a significant difference in the composition of the gut virome, suggesting that even within IBD the phageome can be influenced by environmental changes specific to each disease (Norman et al., 2015). Taken together, this evidence suggests that phages play a role in IBD, particularly within UC and CD. However, the difficulty in moving from correlation to causation in the disease pathology warrants further research into the role of phages as drivers of human diseases.

### Diabetes

Diabetes is a group of diseases characterised by hyperglycemia, caused by a defect in the action of insulin, the secretion of insulin, or a combination of both (American Diabetes Association, 2009). Type 1 diabetes (T1D) is caused by the autoimmune destruction of pancreatic b-cells, whereas Type 2 diabetes (T2D) is a result of insulin resistance (American Diabetes Association, 2009). The gut environment has been associated with the onset of both T1D and T2D, as environmental factors within the gut are thought to act as triggers of disease development.

In patients with T1D a comparison of stool samples to healthy controls showed that the evenness of two major phage groups, *Myoviridae* and *Podoviridae*, was lower, suggesting that the distribution of gut phage is disturbed in individuals with T1D (Zuo et al., 2019). Amyloid-producing *E. coli* present in the gut have been linked to the development of autoantibodies contributing to T1D (Tetz et al., 2019). Diversity of intestinal *E. coli* phages was found to be significantly higher in T1D patients than in control individuals, with almost all the identified *E. coli* phages being lysogenic (Tetz et al., 2019). The ratio of phages to *E. coli* was also shown to increase in those with T1D, suggesting continual induction of prophages, or *E. coli* harbouring prophages may have a fitness advantage in T1D (Tetz et al., 2019). *Bacteroides dorei* has also been found at higher abundance in individuals with T1D and those at risk of developing islet autoimmunity (Leonard et al., 2014). There are a number of methylase genes present in the genomes of *B. dorei* temperate phages, which raises questions about the impact of methylation and prophages on the development of islet autoimmunity (Leonard et al., 2014). In samples from T1D patients, *CrAssphage* were shown to correlate with *B. dorei*, but no other *Bacteroides* (Cinek et al., 2017). However, no significant relationship between the presence of *CrAssphage* and the onset of islet autoimmunity was identified (Cinek et al., 2017).

Compared to healthy control samples, significantly more phages were present in the gut of individuals with T2D, which showed an increased abundance of intestinal *Podoviridae* predicted to infect *Escherichia* and *Clostridium* (Breitbart et al., 2018). As *Clostridium* spp. and *E. coli* have been found to be present in higher numbers in T2D patients, this could suggest that these are temperate phages which proliferate alongside their host bacteria in accordance with the development of T2D (Qin et al., 2012). T2D patients’ gut phages carried a large number of genes which could impact the human host (Ma et al., 2018), suggesting that phages themselves could drive the pathogenesis of human diseases.

Interestingly it has been demonstrated that fecal virome transplant (FVT) can alleviate obesity and T2D in a mouse model, significantly altering both the bacterial and viral components of the gut microbiota (Rasmussen et al., 2020). FVT in mice alleviated some effects of a high-fat diet and normalised blood glucose tolerance, which was not observed in control mice (Rasmussen et al., 2020). It has also been suggested that both *Caudovirales* richness and the overall viral diversity in donor faeces can influence the outcome of FVT in T2D, warranting further investigation (Zuo et al., 2018; Park et al., 2019).

Despite the research to date, the question remains whether gut phages influence the onset and development of T1D and T2D, or whether they act as disease biomarkers, revealing the effects of diabetes on the gut microbiome. The role of temperate phages in human disease is currently unknown and it is yet to be discovered if prophages are induced in the gut environment and drive the development of T1D or T2D, or if prophage induction is a response to autoimmunity.

### Human Immunodeficiency Virus (HIV)

HIV is a highly complex disease with wide-ranging systemic consequences for the patient, even with the use of antiretrovirals. The interaction of the gut, blood, and immune system complicates investigation, but it appears that phages may predict disease progression and influence responses to therapy. HIV infections lead to a depletion in CD4+ T cells, rendering the body vulnerable to infection and disease. The advanced stages of HIV infection are referred to as acquired immunodeficiency syndrome (AIDS), a life-threatening condition associated with excess mortality.

HIV infections initially begin in the gut, with the virus infecting the intestinal-associated lymphoid tissue (Dillon et al., 2016). Research over recent years has questioned the role of the intestinal microbiota in HIV infections, given that these infections are associated with translocation of the microbiota and its products across the damaged intestinal epithelial layer (Brenchley et al., 2006; Klase et al., 2015). The mechanisms by which HIV alters the gut microbiota and how the microbial population of the gut modulates differential responses to infection have previously been reviewed in detail (Dinh et al., 2015; Zilberman-Schapira et al., 2016).

Comparisons of the microbiota of HIV-infected individuals, those receiving antiretroviral treatment, or with varying CD4+ T cell levels found no significant difference in phage richness or diversity (Monaco et al., 2016). In contrast, other studies have observed a significant increase in the number of phage sequences in Simian immunodeficiency virus (SIV)-infected rhesus monkeys, a commonly used animal model of HIV (Handley et al., 2012). As phages have been shown to directly interact with and modulate immune cells, the contribution of phages to HIV infection should be investigated further (Foca et al., 2015).

The plasma of HIV/AIDS patients was dominated by phages and bacteria similar to those found in the human gut, whereas healthy controls showed no detectable phages in their plasma virome (Li et al., 2012). A high proportion of this HIV-associated plasma virome was composed of *Enterobacteria* and *Pseudomonas* phages (Li et al., 2012). This could suggest that phages and bacteria leak from the gut during HIV infection and move into systemic tissues.

The antiretroviral treatment for HIV could potentially have unintended effects on the phageome. An increase in gut dysbiosis has been reported upon administration of antiretroviral therapy, indicating possible consequences for gut bacteria and their phages (Klase et al., 2015). If the gut phage populations differ in HIV-infected individuals, this could have implications for their response to therapy. The ability of phages to influence the leaky gut (Li et al., 2012) must also be considered, as it has been shown that HIV patients with increased gut permeability may not respond to antiretroviral therapy (Marchetti et al., 2008).

### Cardiovascular Disease (CVD)

CVD is a broad term used to define several diseases affecting the cardiovascular system, including ischaemic heart disease, myocardial infarction, and congestive cardiac failure. The driving forces behind the development of CVD are atherosclerosis and hypertension, which are themselves intricately related. Atherosclerosis is an inflammatory response characterised by macrophage activation and Th1 responses, among many other contributing factors. The aetiology of CVD can be bacterial, with direct links between the bacterial metabolites and risk of atherosclerotic cardiovascular disease (Wang et al., 2011).

Changes in the gut phageome are more common in patients with CVD and hypertension (Jie et al., 2017; Han et al., 2018). The presence of chronic inflammation is a driver of atherosclerosis and is seen in many autoimmune diseases (Yarur et al., 2011; Escarcega et al., 2018), and some phages may have a contributory effect. Phages can induce the production of IFN-γ, a key cytokine in macrophage activation. An increase in serum phage load was demonstrated in patients with CVD which potentially plays a role in the driving inflammatory response of the disease (Dinakaran et al., 2014). Oral administration of phage cocktails has been shown to increase gut permeability (Tetz et al., 2017). Subsequently, gut permeability can lead to increased bacteria and bacteriophages in the bloodstream, with potential synergistic effects in congestive cardiac failure (Sandek et al., 2007). To complicate this effect, different phage cocktails induce varying cytokine profile alterations. Oral administration of T7 phages increased serum cytokines such as IL-1α, IL-1β IL-2, IL-12 and IL-17 (Park et al., 2014). However, the role of such cytokines in cardiovascular disease is controversial with evidence of both pro- and anti-atherosclerotic effects (Gong et al., 2015; Williams et al., 2019). Therefore, the role of phages in the development of cardiovascular disease may be due to modulatory effects on the inflammatory response.

Conversely, there is evidence that phages may have a protective role against CVD. Swinepox virus (a eukaryotic virus) has been shown to reduce the incidence of restenosis following stent insertion, a common treatment option for patients with angina pectoris and myocardial infarction (Shimamura et al., 2012). If this is true of eukaryotic viruses, there are potentially similar effects with bacteriophages also. Phage as medical tools, such as phage display, can be utilised for CVD diagnostics (Park et al., 2010) and drug delivery (Nicol et al., 2009). Phage therapy can be used to prevent or treat post-heart surgery infections (Potapov et al., 2020), as well as altering the detrimental metabolism of bacteria in high-risk patients.

As CVD is complex in its aetiology, it is difficult to define the specific role phages may play in the disease. However, it seems clear that phage communities vary between healthy and CVD populations, in addition to their immunomodulatory role, suggesting this area is worthy of further investigation.

### Autism Spectrum Disorder (ASD)

ASD is a developmental disorder characterised by significant variation in social, communication and behavioural traits. Gut microbiota changes have been observed in patients with ASD, typified by reduced biodiversity and changes in gut anaerobic bacteria abundance, potentially contributing to the disorder’s severity (Parracho et al., 2005; Krajmalnik-Brown et al., 2015).

An alteration in gut permeability is a hypothesis for the development of several diseases, including ASD, and the effects of phages on the gut microbiome may provide a mechanism for this (Tetz and Tetz, 2016). A trail of a modified FMT treatment showed gut bacterial populations of individuals with ASD became more similar to healthy populations, whereas phage populations remained more diverse; these changes were linked with an improvement in ASD symptoms (Kang et al., 2017). The gut phageome is known to change through childhood (see section *Development of the Phageome*) (**Figure 3**), and it is possible that gut dysbiosis during childhood may contribute to the development or severity of ASD, as well as several other psychiatric disorders (Lim et al., 2016; Sherwin et al., 2016; Dickerson et al., 2017). For example, *Lactobacillus* phage φadh has been found to be more common in the oropharyngeal microbiome in patients with schizophrenia (Sherwin et al., 2016).

Psychiatric disorders are extremely complex, with the direct causes remaining unknown. From current evidence it appears that phages, as part of the larger gut microbiome community, are likely to play a role at some level, which may become clearer with future research.

### Does the Phageome Play a Role in Human Gut Health?

An increase in the abundance of gut phages appears to be a common theme across the diseases reviewed here (**Table 6**). There is a general shift in the phageome in CD and UC to an increased number of *Caudovirales*, with an expansion of a few particular phage species (Lepage et al., 2008; Wagner et al., 2013; Norman et al., 2015; Fernandes et al., 2019; Zuo et al., 2019). However, studies of the phageome in other human diseases supported evidence that an increase in specific phage species correlated with disease (Li et al., 2012; Breitbart et al., 2018; Tetz et al., 2019). This could suggest that the role of phages in human disease may be specific to particular gut phage-host relationships, rather than an overall trend within the entire gut phage community.

Another common theme is a strong association with dysregulated inflammation underlying disease, which potentially could elicit phageome’s influence on human disease. Phage proteins can directly induce gut inflammation (Norman et al., 2014; Majewska et al., 2015; Miernikiewicz et al., 2016), but whether this is a response to changes in the gut microbiome, or a driving factor of gut dysbiosis, is yet to be determined.

Bacterial communities of the gut are the most studied for their impact on human health and disease, recent research has further highlighted other populations in the gut communities, including fungal (Nash et al., 2017) and archaeal communities (Dridi et al., 2011). Research into the phageome, to date, has aimed to understand fluctuations in the abundance and diversity of phage groups, and how these may correlate with disease. However, we are yet to identify any specific functional relationships between phages and human disease. Moving forward, research into the mechanisms by which the gut phageome influences disease is necessary and may help answer a number of vital questions. Are specific phage species involved in causing certain health conditions, or is disease influenced by the dynamics of the phageome as a whole? What precise biological mechanisms are behind phage immunomodulation? Are phages the driving force behind disease, or are fluctuations in the individual phageome a result of changing gut bacterial populations? The conditions discussed in this review are complex and are often associated with individual genetics combined with environmental triggers; how does the impact of the phageome vary between individuals in the context of disease? Resolving these questions will provide a clearer view of the functional relationship between the phageome and human disease.

Research so far has presented data on how both phage and bacterial populations change in comparison to healthy controls. While this has highlighted areas in which these microbial gut communities influence disease, we are not frequently given a longitudinal perspective about how these communities change with progression from a healthy state to diseased. As earlier stated (see section *Impact of Medical Interventions on the Phageome*), there is limited scope for establishing what can be described as a “core phageome”, compared to our knowledge of gut bacterial populations. Variation between individuals is great, and therefore elucidating if changes in phage populations are due to a single factor is difficult. Longitudinal studies are becoming more common, with metagenomic studies of UC and CD expanding, which may help to define a core phageome and establish biomarkers of disease (Norman et al., 2015; Manrique et al., 2016; Clooney et al., 2019). However the chicken-or-egg argument remains; which came first, the changes in the microbiome or did the microbiome adapt to the disease? The phageome adds another layer of complexity to this discussion, did the phageome, bacterial host or human disease initiate these changes? By using model systems such as mouse studies, we may be able to more easily manipulate these factors to unpick the true workings of the gut phageome. However, reducing gut phageome studies to these less complex systems eliminates the influence of the multifaceted human life and cannot be fully reflective of the true workings of the phageome.

Despite the microbiome receiving much more in-depth research than the phaegome, limited applications to prevent or treat diseases exist (Durack and Lynch, 2019). Therefore, it is likely to be a long time before any therapeutic options for curing systemic disease by altering the phageome become available. Perhaps understanding any perturbations of the phageome and microbiome following the application of a medical treatments, such as phage therapy, would provide insight into potential new therapies focussed on the phageome.

## Conclusions

In this paper we have shown there is a wealth of literature on the role of bacteriophages within the human gut. However, there are still large areas which remain unknown or complex, which with further investigation could lead to discoveries that inform beneficial treatments for human disease, in a similar fashion as we have seen with the microbiome.

The methods used to study phages influence the communities detected. With recent advances in technology studying in-depth metagenomes, it is becoming easier to elucidate how these viral populations change and are influenced by their environment. A prominent example of these new discoveries are *CrAssphage*, recent work has highlighted its existence as an important component of the human gut phageome. These are just one group out of many phages in the diverse populations of microorganisms that colonize our gastrointestinal tract from birth.

We are discovering that phage populations are highly specific to individuals and the existence of a core phageome, like the microbiome, is a loose term. Phages perform a number of functions in the human gut including: genetic exchange, maintenance of diversity, supply of nutrients and interactions with the immune system. Additionally, host and virus dynamics are highly complex and vary depending on the context in which predator and prey or symbionts meet. Although an understudied area, it does appear that phage populations vary between healthy individuals and disease states. It is well understood that the micro- and mycobiomes influence a variety of human conditions; this also applies to the phageome. Further research is warranted to establish if phage manipulation would be beneficial in these conditions.

From the evidence discussed in this paper, we believe that the role and impact of the phageome is underexplored, and with continued investigation potential therapies and a deeper understanding of the viral influence on human health can be discovered.

### Perspectives and Future Directions

Since the importance of the gut microbiome has come to light over the past decade, we have increased our understanding of the function of the human body and how it is not solely “human”. The reliance we have on microbial communities for even our basic biology has become abundantly clear. However, as discussed throughout this paper, the largely overlooked phageome evidently plays a major role.

We hope that with the continuing improvements in the availability and accessibility to genomic research methods, the workings of the phageome will be increasingly understood as we have seen with the microbiome. As discussed in this paper, DNA technologies are often utilised, but RNA techniques are still lacking. Unfortunately this leaves a portion of the phageome without proper characterisation.

Discovering how these communities interact and interplay with their human host may lead to improvements in health, nutrition and wellbeing through manipulation of these microscopic communities. We believe this is of heightened importance in light of increasing antimicrobial resistance as traditional antibiotic therapies may become redundant. Phage therapy has already been established as a viable alternative treatment and has found a recent resurgence in popularity in Western medicine. We hope this therapy could be applied in complex diseases such as those discussed in this paper, in which the gut microbiota is disturbed. Understanding more about the phageome will in turn further our knowledge of human health, leading to a brighter future for human health.

## Author Contributions

EJ conceived, designed and critically reviewed the manuscript. The majority of the manuscript was written by ET, while LK, GM, JB, NH, and DL contributed specialist sections. The figures and tables were compiled by EJ, ET, LK, GM, NH, and DL. All authors contributed to the article and approved the submitted version.

## Funding

This work was supported by Warwick Integrative Synthetic Biology (WISB), funded jointly by BBSRC/EPSRC, grant ref: BB/M017982/1 under the UK Research Councils’ Synthetic Biology for Growth programme. The work has also been supported by PhD fellowships awarded to LK, GM, and JB through DTPs funded by BBSRC, EPSRC and NERC.

## Conflict of Interest

The authors declare that the research was conducted in the absence of any commercial or financial relationships that could be construed as a potential conflict of interest.
